# The unprecedented era of aging

**DOI:** 10.1186/s41232-019-0104-2

**Published:** 2019-08-01

**Authors:** Yuki Sato, Motoko Yanagita

**Affiliations:** 1 Department of Nephrology, Graduate School of Medicine, Kyoto University, 54 Shogoin-Kawahara-cho, Sakyo-ku, Kyoto, 606-8507 Japan; 2 Medical Innovation Center TMK Project, Graduate School of Medicine, Kyoto University, Kyoto, Japan; 3 Institute for the Advanced Study of Human Biology (ASHBi), Kyoto University, Kyoto, Japan

Advances in medical care have dramatically expanded individual life expectancy and the global population is aging. By 2050, the world population over 60 years will exceed the size of the global population of young individuals and the world population over 80 years will become more than triple, reaching 380 million individuals [1]. As the population ages, the prevalence of aging-associated diseases will be also increased, resulting in the rise of the global burden of disease and disability. Therefore, understanding mechanisms for healthy aging is of significant importance.

Because aging is the major risk factor for a variety of chronic diseases, including cardiovascular diseases, cancer, and metabolic diseases, which lead to the decreased quality of life, frailty, and mortality among the elderly, therapeutic strategies targeting aging pathways are receiving more attention and are subjects of intense research. For instances, with aging, immune system undergoes a broad range of functional alterations, globally termed as immunosenescence. Inflammaging, a systemic low-grade inflammation that occurs during aging, is one of the most characteristic traits of immunosenescence and has been speculated as a common pathophysiology underlying various types of age-related diseases described above. Actually, a recent success of the Canakinumab Anti-inflammatory Thrombosis Outcome Study (CANTOS) trial, which showed that IL-1β inhibition has potential to improve outcomes for patients with established coronary artery atherosclerosis, highlighted the relevance of targeting the common age-dependent pathology [2].

However, the story of aging is not so simple. Several lines of evidence have also reported that loss of function in old age have profound roots into the early events of life, and some interventions to delay the onset or progression of age-associated diseases should be scheduled when people are still young. Additionally, the biological aging and chronological aging is not always the same, and the importance of distinguishing between them is noted for better understanding in the pathophysiology of age-related diseases. Although aging processes greatly vary between individuals, genders, and genetic backgrounds, and are modified by environmental factors throughout life, their variations and drivers are just beginning to be clarified.

In this thematic series reviews, we invited the leading researchers on this aging research field. Dr. Fukushima and colleagues reviewed their series of original studies regarding on senescence-associated T cells (SATs), a unique age-dependent CD4 memory T cell subset with cellular senescence phenotype. Development of SAT is closely related with thymic involution occurred during puberty, and SAT has been implicated in several pathological conditions. Dr. Kishimoto and colleagues summarized regulation of aging and roles of environmental factors on aging, with a focus on the studies in the nematode *Caenorhabditis elegans*, and emphasized that aging is a complex phenotype that is regulated by intrinsic and extrinsic influences throughout life. Here, we would like to express sincere appreciation to the distinguished researchers who contributed to this special issue and sincerely hope that these review articles will provide novel insights to the researchers in the broad field of inflammation and regeneration.

## References

[1] Harper S (2014). Economic and social implications of aging societies. Science.

[2] Ridker PM (2017). Antiinflammatory therapy with canakinumab for atherosclerotic disease. N Engl J Med.

